# The Effect of Bone Morphogenetic Protein 2 or Extracellular Signal-Regulated Kinase 1 Silencing on Phosphorus Utilization and Related Parameters in Primary Broiler Osteoblasts

**DOI:** 10.3389/fvets.2022.943864

**Published:** 2022-06-30

**Authors:** Yanqiang Geng, Tingting Li, Yun Hu, Liyang Zhang, Xiaoyan Cui, Ling Zhu, Bingxin Wu, Xugang Luo

**Affiliations:** ^1^Poultry Mineral Nutrition Laboratory, College of Animal Science and Technology, Yangzhou University, Yangzhou, China; ^2^Mineral Nutrition Research Division, Institute of Animal Science, Chinese Academy of Agricultural Sciences, Beijing, China

**Keywords:** chick (Gallus gallus), primary osteoblast, BMP2, ERK1, silencing, P utilization

## Abstract

Two experiments were conducted to study the effect of bone morphogenetic protein 2 (BMP2) or extracellular signal-regulated kinase 1 (ERK1) silencing on phosphorus (P) utilization and related parameters in primary broiler osteoblasts. Experiment 1 was carried out to select the most efficacious siRNAs against BMP2 or ERK1 for the subsequent experiment. In experiment 2, with or without the siRNA against BMP2 or ERK1, primary broiler osteoblasts were incubated in the medium supplemented with 0.0 or 2.0 mmol/L of P as NaH_2_PO_4_ for 12 days. The osteoblastic P utilization and related parameters were determined. The results showed that the si980 and si1003 were the most effective (*P* < 0.05) in inhibiting BMP2 and ERK1 expressions, respectively. The BMP2 silencing reduced (*P* < 0.004) the osteoblastic P retention rate, alkaline phosphatase (ALP) activity, BMP2 mRNA and protein expressions. Supplemental P increased (*P* = 0.0008) ALP activity. Significant interactions (*P* < 0.04) between the gene silencing and supplemental P level were observed in both mineralization formation and bone gal protein (BGP) content. The BMP2 silencing decreased (*P* < 0.05) mineralization formation at both 0.0 and 2.0 mmol/L of added P levels, but the decreased degree was greater at 2.0 mmol/L of added P level, while BMP2 silencing reduced (*P* < 0.05) BGP content at only 2.0 mmol/L of added P level. The ERK1 silencing decreased (*P* < 0.004) mineralization formation, ALP activity, BGP content, *ERK1* mRNA, ERK1 and p-ERK1 protein expressions. Supplemental P increased (*P* < 0.03) mineralization formation, ALP activity, BGP content and p-ERK1 protein expression, but inhibited (*P* = 0.014) ERK1 protein expression. There was an interaction (*P* < 0.03) between the gene silencing and supplemental P level in the osteoblastic P retention rate. The ERK1 silencing decreased (*P* < 0.05) it regardless of 0.0 or 2.0 mmol/L of added P level, but the reduced degree was greater at 2.0 mmol/L of added P level. It was concluded that either BMP2 or ERK1 silencing suppressed P utilization, and thus either of them participated in regulating P utilization in primary broiler osteoblasts.

## Introduction

The studies of molecular mechanisms of osteogenic key gene functions in regulating phosphorus (P) utilization in primary broiler osteoblastic models will help to reduce poultry production costs and environmental concerns. The genes of bone morphogenetic protein 2 (BMP2) and activating extracellular signal-regulated kinase 1 (ERK1) play a crucial role in regulating osteoblastic differentiation and P homeostasis (1–4). Alkaline phosphatase (ALP) and bone gal protein (BGP) are the important regulators of bone matrix mineralization and adjust the hydroxyapatite formation (3). The BMP2 is the most potent inducer for the osteoblastic mineralization and stimulating the osteoblast differentiation. Earlier researches indicated that BMP2 was involved in bone signal responses and promoted the expressions of ALP and BGP and osteoblastic mineralization via ERK1 (5–9). Sun et al. (3) reported that overexpression of BMP2 increased the formation of mineralization nodules and promoted ALP activity in bone mesenchymal stem cells of rats. Furthermore, ERK1 inhibitor was shown to suppress the ALP activity, bone mineralization and downregulate the expression of BMP2 mRNA in mice bone mesenchymal stem cells (10). The results from our studies *in vivo* and *in vitro* have demonstrated that P utilization in the bone and osteoblasts of broilers might be partially regulated by BMP2 and ERK1 (11, 12). However, it is unknown if BMP2 or ERK1 is directly involved in regulating P utilization in primary broiler osteoblasts. Therefore, we hypothesized that either BMP2 or ERK1 silencing would affect P utilization in primary broiler osteoblasts. The objective of the present study was to investigate the effect of either BMP2 or ERK1 silencing on P retention rate, mineralization formation, ALP activity and BGP content in primary broiler osteoblasts in order to test the above hypothesis.

## Materials and Methods

The primary cultured osteoblasts of broiler chicks were used in the present study, and the Animal Care Committee of Yangzhou University approved the care and handling of the broiler chicks from which the tibial osteoblasts were obtained.

### Reagents

Dulbecco's Modified Eagle's Medium (DMEM) and 0.25% trypsin were purchased from Maichen Technology (Beijing, China). Dulbeccos phosphate-buffer saline (D-PBS), 5,000 U mL^−1^ penicillin, 5,000 μg mL^−1^ streptomycin, fetal bovine serum (FBS), P-free DMEM and L-glutamine were purchased from Thermo Fisher Scientific (Waltham, USA). The 1% Alizarin Red S (ARS) was purchased from Solarbio Technology (Beijing, China). The ALP and BGP assay kits were purchased from Nanjing Jiancheng Bioengineering Institute (Nanjing, China). Dexamethasone and ascorbic acid were purchased from Sigma-Aldrich (Louis, USA). Acetic acid glacial and ammonium hydroxide were purchased from Shanghai Macklin Biochemical Co., Ltd (Macklin, China). The JetPRIME reagent was purchased from Polyplus-transfection^®^ SA (Strasbourg, France).

### Isolation, Culture, and Treatments of Primary Broiler Osteoblasts

The primary osteoblasts were isolated by tissue explant method, and their culture and treatments were done as previously described (13). Briefly, the tibiae were obtained from one-day-old chicks (male Arbor Acres broilers, purchased from Jinghai Poultry Group Co., Ltd., Jiangsu, China), scraped cleanly, and then cut into 1 mm^2^ pieces. Whereafter, the clean pieces were incubated in DMEM with 15% FBS, 1% penicillin/streptomycin and 1% L-glutamine at 37 °C in a humidified atmosphere containing 5% CO_2_. Once the cells migrated from bone pieces and reached to confluency, the cells were digested with 0.25% trypsin and then sub-cultured in 6-well-plates (5 × 10^5^ cells/well) in the above DMEM complete medium. When the sub-cultured cells grew to 80–90% confluency, different treatments were performed.

### Design and Synthesis of siRNAs

The siRNAs directed against BMP2 (namely si771, si988 and si1125) or ERK1 (namely si610, si936 and si1003) were designed (BMP2, NM_204258; ERK1, NM_204150.1). One negative control siRNA (NC-siRNA), which did not target any known chicken genes, was also designed. All of the siRNAs (Table 1) were synthesized by the Suzhou Genepharma Co., Ltd (Jiangsu, China).

**Table 1:** Sequences of the synthesized siRNA molecules and their positions in genomic regions of target genes (experiment 1).

**Target genes**	**siRNAs**	**Sequences (5'-3')**	**Genomic positions**
*BMP2*	si771	Sense: GCCGUUGUUAGUGACGUUUTT Antisense: AAACGUCACUAACAACGGCTT	771–791
	si988	Sense: GCAGAUCACCUAAACUCAATT Antisense: UUGAGUUUAGGUGAUCUGCTT	988–1,008
	si1125	Sense: GGUCGUACUAAAGAACUAUTT Antisense: AUAGUUCUUUAGUACGACCTT	1,125–1,145
*ERK1*	*si*610	Sense: CCUGAAAUCAUGCUGAAUUTT Antisense: AAUUCAGCAUGAUUUCAGGTT	610–630
	si936	Sense: GGAGCAAGCUUUAGCCCAUTT	936–956
		Antisense: AUGGGCUAAAGCUUGCUCCTT	
	si1003	Sense: GCACCCUUCAAGUUUGAUATT	1,003–1,023
		Antisense: UAUCAAACUUGAAGGGUGCTT	
Negative control	NC-siRNA	Sense: UUCUCCGAACGUGUCACGUTT Antisense: ACGUGACACGUUCGGAGAATT	

*BMP2, bone morphogenetic protein 2; ERK1, extracellular signal-regulated kinase 1*.

### Screening of the Most Effective siRNAs Against BMP2 or ERK1 (Experiment 1)

To screen the most effective siRNA against BMP2 or ERK1, the cells were randomly divided into one of eight treatments with three replicates per treatment, including the blank control group (CON), negative control (NC) group, si771 group, si988 group, si1125 group, si610 group, si936 group and si1003 group. The cells were transfected with 80 pmol/well-siRNA (NC-siRNA, si771, si988, si1125, si610, si936, or si1003) using the JetPRIME reagent according to the manufacturer's instructions, and the CON cells were not treated at all. At 48 h of post-transfection, the cells were collected for evaluating the suppressive effects of the siRNAs against BMP2 or ERK1 by using real time quantitative PCR (RT-qPCR) and Western blotting. Either the BMP2-specific siRNAs or ERK1-specific siRNAs shared the same controls (CON and NC). Among the siRNAs we examined, si988 and si1003 were, respectively screened as the most powerful siRNAs for BMP2 and ERK1 suppressions.

### Effect of BMP2 or ERK1 Silencing on P Utilization and Related Parameters in Primary Broiler Osteoblasts (Experiment 2)

A completely randomized design involving a 2 × 2 factorial arrangement with two added P levels (0.0 and 2.0 mmol/L of P as NaH_2_PO_4_) and two cell types (normal cells and BMP2 or ERK1 silencing cells). There were 4 replicates for each treatment. The normal cells were transfected with the NC-siRNA, and the BMP2 or ERK1 silencing cells were, respectively transfected with si988 and si1003. At 24 h post-transfection, all cells were washed twice with the P-free DMEM medium to remove the residual P. Then, the normal cells and BMP2 or ERK1 silencing cells were incubated in the osteogenic induction medium (OIM) supplemented with 0.0 or 2.0 mmol/L of P for 12 days. Selecting the supplemental P dose of 2 mmol/L in the present study was based on our previous studies (11, 13). The OIM was the P-free DMEM supplemented with 15% FBS, 1% penicillin/streptomycin, 1% L-glutamine, 100 nmol/L Dex and 50 μg/mL ascorbic acid. Without and with supplemental P, the mediums were analyzed to contain 0.68 and 2.57 mmol/L of total P, respectively. Furthermore, during the period of P treatment, transfection was performed every 3 days until samples needed to be collected. During the P treatment period, the old medium was collected and pooled together at every medium change to determine the osteoblastic P retention rate. At the end of P treatment, the cells were collected for analyses of P utilization-related parameters (mineralization formation, ALP activity and BGP content).

### Osteoblastic P Retention Rate Determination

The total P contents in the fresh or old medium were determined as previously described (13). Briefly, 1 mL of fresh or old medium was digested with HNO_3_-HClO_4_ (5:1) of mixed acid at 200 °C. The digested residues were dissolved in 10 mL of 2% HNO_3_ and then used for the total P determination. The osteoblastic P retention rate (%) = (V1 × C1-V2 × C2)/V1 × C1, where V1 and C1 represent, respectively total volume (mL) and total P content (mmol/L) of the added fresh medium; V2 and C2 represent, respectively total volume (mL) and total P content (mmol/L) of the pooled old medium.

### Detection and Quantification of Mineralization Formation

The mineralization formation was quantified by ARS straining as previously described (14). Briefly, the cells were firstly strained with 1% ARS (pH 4.2) for 10 min. After drying, the content of each well was then solubilized with 10% (v/v) acetic acid, collected into a tube and heated at 85°C for 10 min. The slurry was centrifuged at 20,000 *g* for 15 min, and the supernatant was neutralized with 10% (v/v) ammonium hydroxide, and then the absorbances read in a 96-well-plate at 405 nm. Finally, the ARS concentrations were calculated based on the ARS standard curve. In the present study, the mineralization formation was represented by the ARS concentration (mmol/L).

### Determinations of ALP Activity and BGP Content

The osteoblastic ALP activity was determined by a microplate reader with commercial kits. The osteoblastic BGP content was determined by the ELISA method with commercial kits.

### RT-qPCR Analysis

The mRNA expressions of *BMP2* and *ERK1* in the osteoblasts were determined by the RT-qPCR according to the procedure as previously described (11). The 2^−ΔΔCt^ method was used to calculate the relative mRNA expressions of target genes to reference genes including β-*actin* and glyceraldehyde-3-phosphate dehydrogenase (*GAPDH*). The primer sequences used in the present study were shown in Table 2.

**Table 2:** Primers used for the target and reference genes (experiment 2).

**Genes**	**Sequence (5'-3')**	**Product length (bp)**	**GenBank ID**
*BMP2*	Forward: GTTTGTGGTGGAGGTGGTTC Reverse: GTCCACATACAACGGATGCC	238	NM_204358.1
*ERK1*	Forward: TGCTTTCCCTACCACACAAA	131	NM_204150.1
	Reverse: AGCTTGCTCCACTTCAATTCG		
β-*actin*	Forward: ACCTGAGCGCAAGTACTCTGTCT Reverse: CATCGTACTCCTGCTTGCTGAT	169	NM_205518.1
*GAPDH*	Forward: CTTTGGCATTGTGGAGGGTC Reverse: ACGCTGGGATGATGTTCTGG	82	NM_204305.1

*BMP2, bone morphogenetic protein 2; ERK1, extracellular signal-regulated kinase 1; GAPDH, glyceraldehyde-3-phosphate dehydrogenase*.

### Western Blotting Assay

Total and phosphorylated protein expression levels of target genes were analyzed by Western blotting as previously described (11, 15, 16). The primary antibodies used in the experiment included anti-BMP2 (catalog no. A0231; Abclonal, Wuhan, China), anti-EKR1 (catalog no. 16443-1-AP; Proteintech, Wuhan, China), anti-phosphorylated EKR1 (catalog no. AP0234; Abclonal, Wuhan, China) and anti β-tubulin (catalog no. HX1829; Huaxingbio, Beijing, China). Horseradish peroxidase (HRP)-conjugated goat anti-rabbit (catalog no. HX2030; Huaxingbio, Beijing, China) and goat anti-mouse (catalog no. HX2032; Huaxingbio, Beijing, China) were used as secondary antibodies. The membranes were then visualized using the ECL system (Tanon, Shanghai, China).

### Statistical Analyses

All statistical analyses were conducted using the GLM procedure of SAS 9.4 (SAS institute Inc., Cary, NC, USA). The data of experiment 1 were subjected to one-way ANOVA, whereas the data of experiment 2 were analyzed by two-way ANOVA, and the statistical model included the added P level, cell type and their interaction. The replicate served as the experimental unit. Different means were separated using the LSD method, and the statistical significance was declared at *P* ≤ 0.05.

## Results

### Interference Efficiencies of siRNAs in Primary Broiler Osteoblasts (Experiment 1)

**Figure 1:**
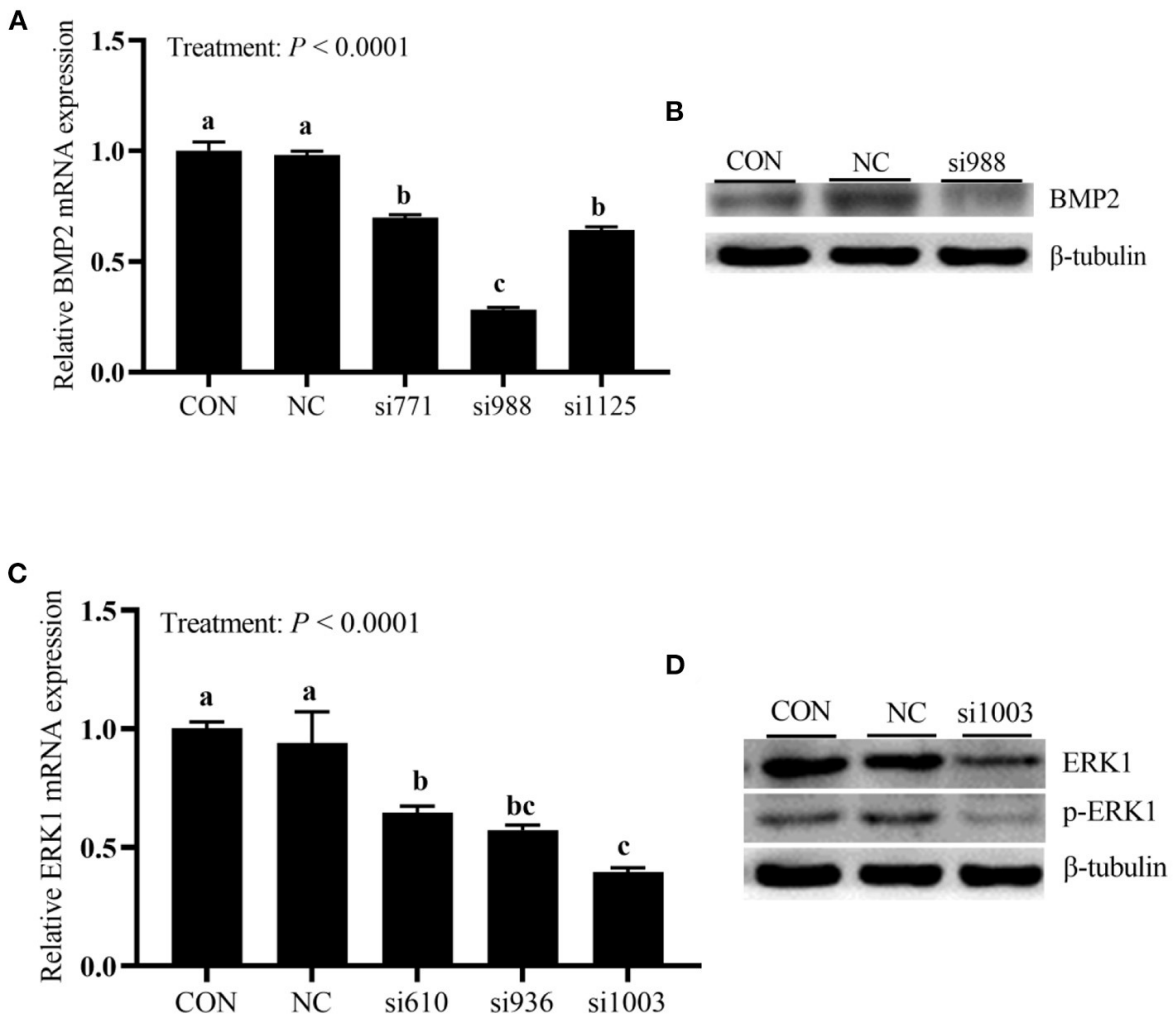
Screening of the most effective siRNAs against *BMP2* mRNA and protein **(A,B)**, and *ERK1* mRNA and protein **(C,D)** in primary broiler osteoblasts (experiment 1). Each value represents the means ± SE, *n* = 3. Lacking the same letters (a, b, c) means significant differences, *P* < 0.05. BMP2, bone morphogenetic protein 2; ERK1, extracellular signal-regulated kinase 1.

As shown in Figure 1A, the treatment affected (*P* < 0.0001) *BMP2* mRNA expression. Compared with the NC and CON groups, the expressions of *BMP2* mRNA in cells transfected with three BMP2-siRNAs decreased (*P* < 0.05). However, the expression of *BMP2* mRNA in the si988 group was the lowest (*P* < 0.05) with 70% of the inhibition efficiency compared with the CON, and no difference (*P* > 0.05) between si771 and si1125 groups was observed. As shown in Figure 1C, when compared with the NC and CON groups, the expressions of *ERK1* mRNA in cells transfected with three ERK1-siRNAs decreased (*P* < 0.05). However, the expression of *ERK1* mRNA was lower (*P* < 0.05) in si1003 group than in the si610 group with 60% of the si1003 inhibition efficiency compared with the CON, and no differences (*P* > 0.05) between si1003 and si936 groups and between si610 and si936 groups were observed. In addition, BMP2, ERK1 and p-ERK1 protein expressions were also clearly inhibited (Figures 1B,D). Taken together, these results indicated that the si988 and si1003 could be selected as the most effective siRNAs for BMP2 and ERK1 to be used in the following experiment 2.

### Effect of BMP2 Silencing on P Utilization and Related Parameters in Primary Broiler Osteoblasts (Experiment 2)

**Figure 2:**
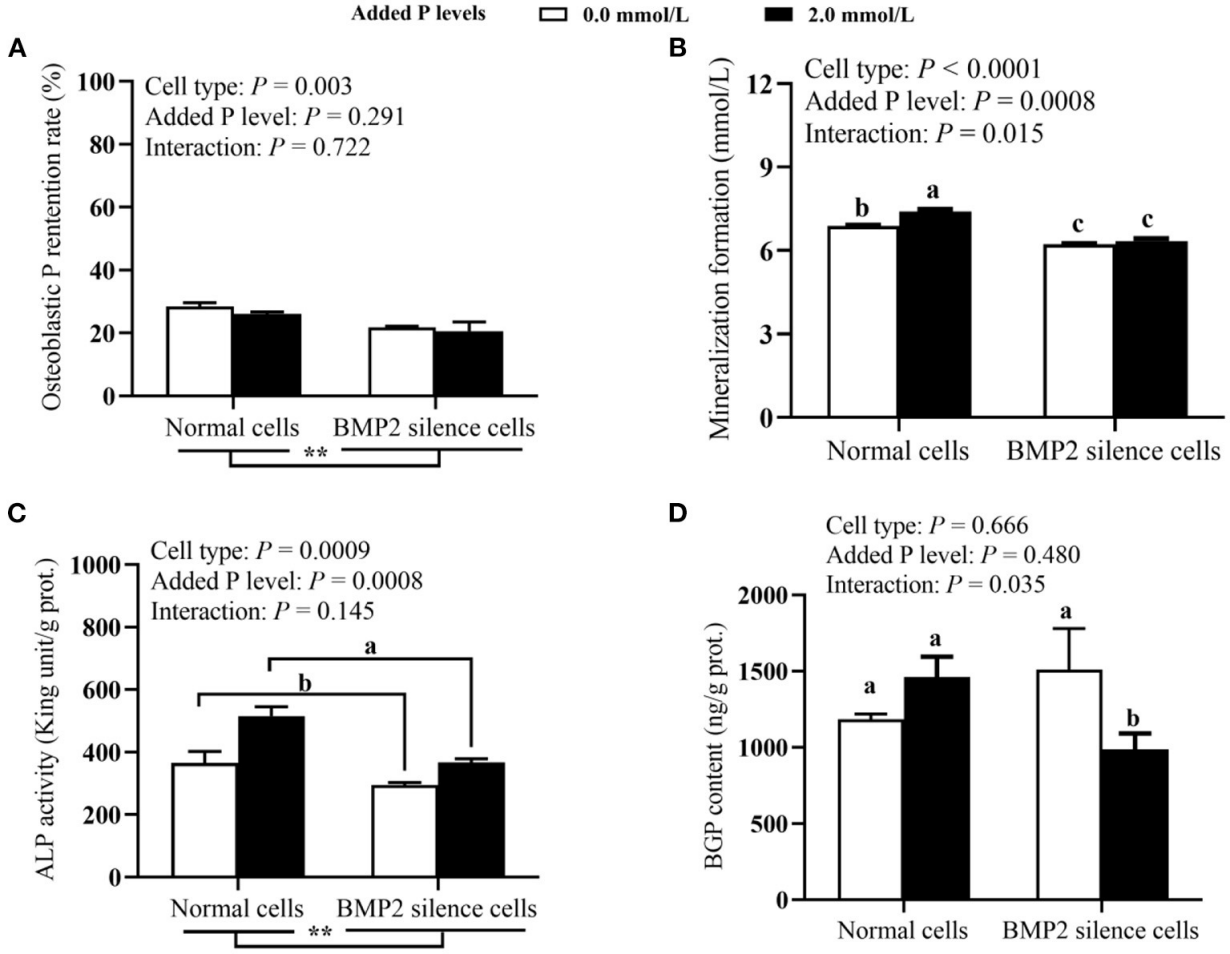
Effects of cell types and P levels on the P retention rate **(A)**, mineralization formation **(B)** ALP activity **(C)**, and BGP content **(D)** in primary broiler osteoblasts (experiment 2). Each value represents the means ± SE, *n* = 4. Lacking the same letters (a, b, c) means significant differences, *P* < 0.05. **P < 0.01. ALP, alkaline phosphatase; BGP, bone gla protein; BMP2, bone morphogenetic protein 2; P, Phosphorus.

**Figure 3:**
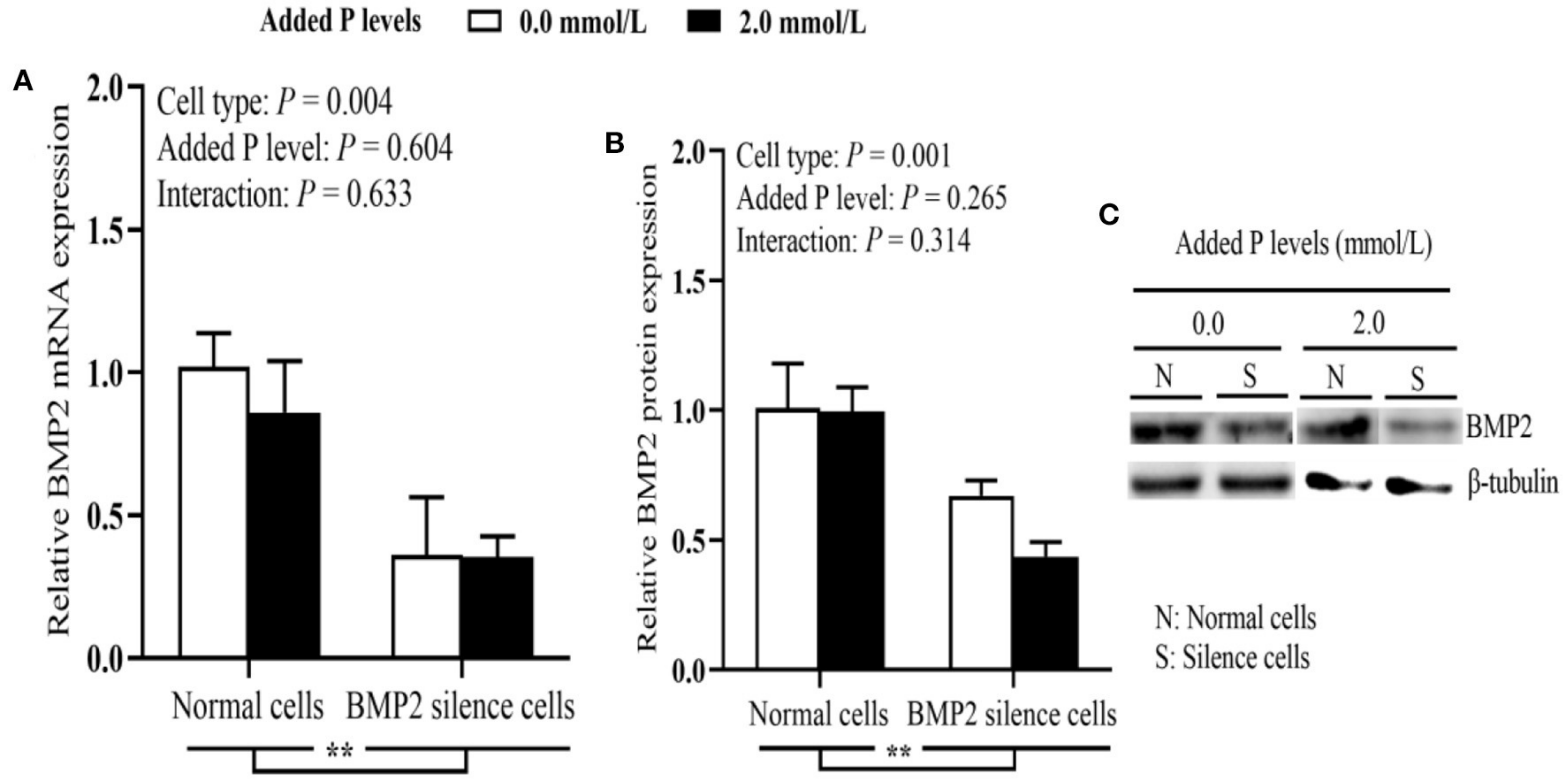
Effects of cell types and P levels on the tibial osteoblastic *BMP2* mRNA **(A)** and protein expressions **(B,C)** in primary broiler osteoblasts (experiment 2). Each value represents the means ± SE, *n* = 4. **P < 0.01. BMP2, bone morphogenetic protein 2; P, Phosphorus.

As shown in Figures 2A–D, the BMP2 silencing reduced (*P* ≤ 0.003) the P retention rate and ALP activity. The osteoblastic ALP activity at 2.0 mmol/L of added P level was higher (*P* = 0.0008) than that at 0.0 mmol/L of supplemental P level. The BMP2 silencing decreased (*P* < 0.05) mineralization formation at both 0.0 and 2.0 mmol/L of added P levels, but the reduced degree was greater at 2.0 mmol/L of added P level. The BMP2 silencing reduced (*P* < 0.05) osteoblastic BGP content supplemented with 2.0 mmol/L P, but had no effect (*P* > 0.05) on it at 0 mmol/L of added P level. As shown in Figures 3A–C, both BMP2 mRNA and protein levels were decreased (*P* ≤ 0.004) in BMP2-silenced cells. However, added P level and their interaction had no effects (*P* > 0.26) on BMP2 mRNA and protein expressions.

### Effect of ERK1 Silencing on P Utilization and Related Parameters in Primary Broiler Osteoblasts (Experiment 2)

**Figure 4:**
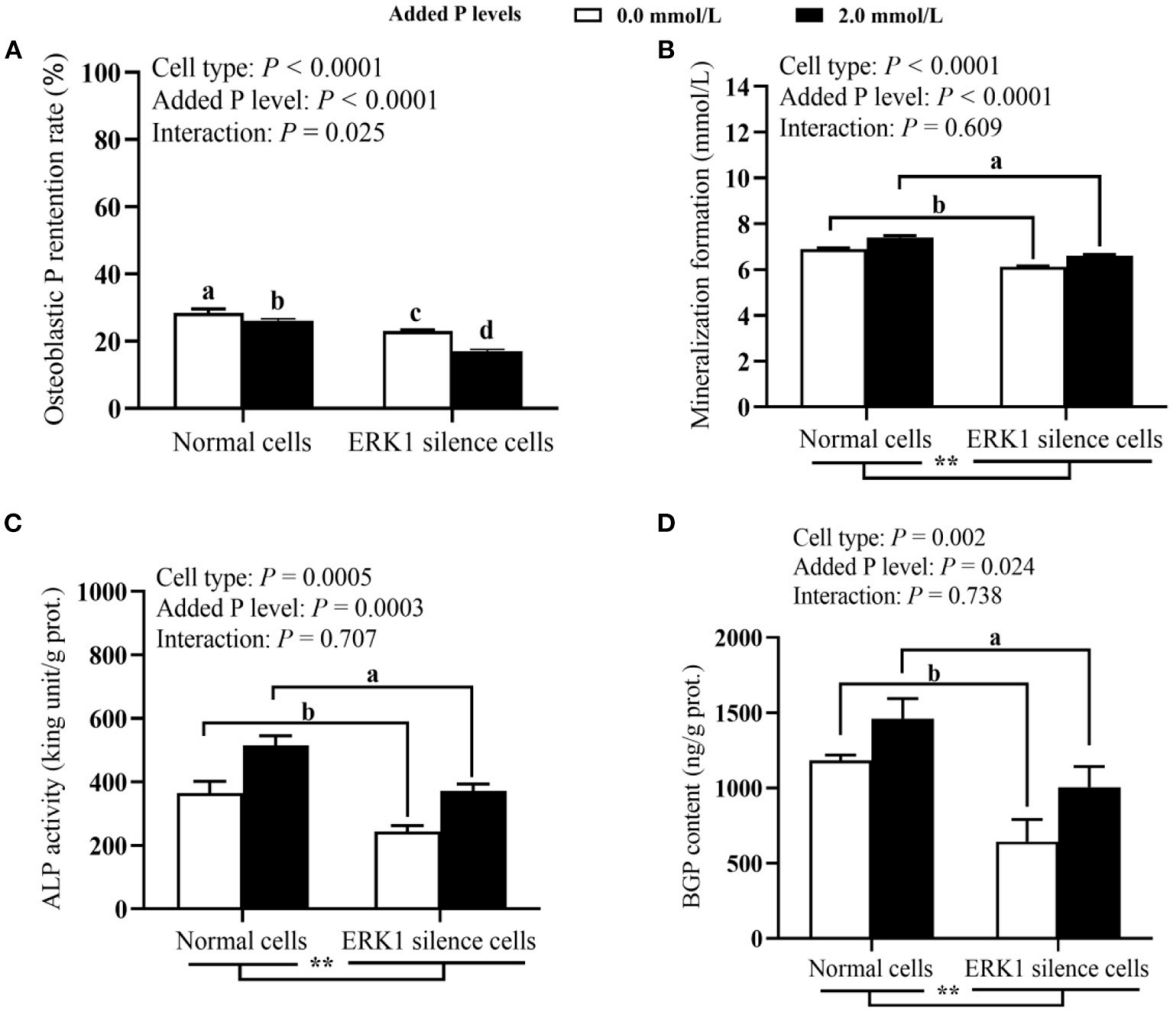
Effects of cell types and P levels on the P retention rate **(A)**, mineralization formation **(B)** ALP activity **(C)**, and BGP content **(D)** in primary broiler osteoblasts (experiment 2). Each value represents the means ± SE, *n* = 4. Lacking the same letters (a, b, c, d) means significant differences, *P* < 0.05. ** *P* < 0.01. ALP, alkaline phosphatase; BGP, bone gla protein; ERK, extracellular signal-regulated kinase 1; P, Phosphorus.

**Figure 5:**
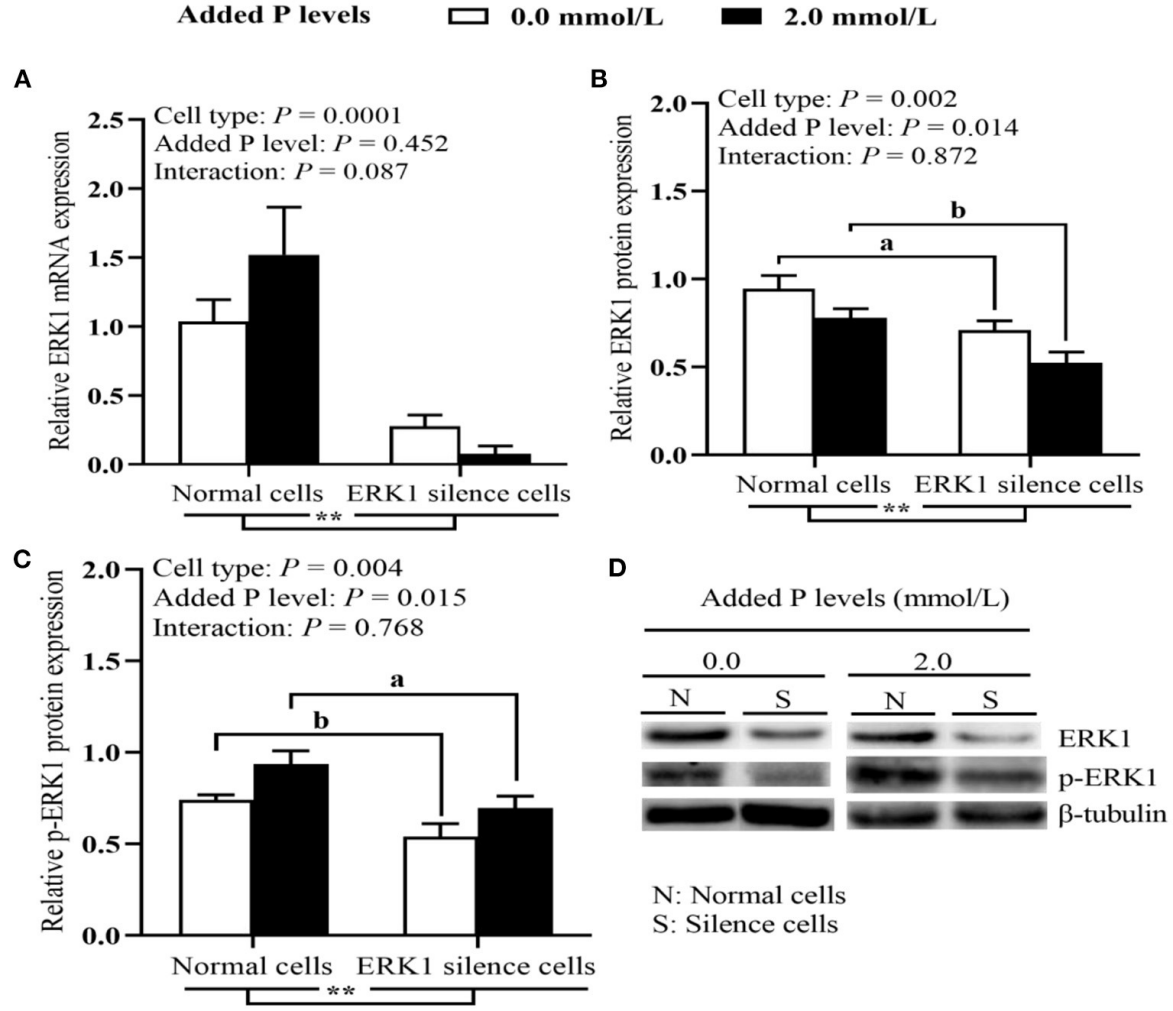
Effects of cell types and P levels on the tibial osteoblastic *ERK1* mRNA **(A)**, ERK1 and p-ERK1 protein expressions **(B–D)** in primary broiler osteoblasts (experiment 2). Each value represents the means ± SE, *n* = 4. Lacking the same letters (a, b) means significant differences, *P* < 0.05. ** *P* < 0.01. ERK, extracellular signal-regulated kinase 1; P, Phosphorus.

As shown in Figures 4A–D, both cell type and added P level had significant effects (*P* ≤ 0.024) on osteoblastic mineralization formation, ALP activity and BGP content. Cell type, added P level and their interaction had significant effects (*P* ≤ 0.025) on P retention rate of osteoblasts. The ERK1 silencing decreased (*P* ≤ 0.002) osteoblastic mineralization formation, ALP activity and BGP content. The osteoblast mineralization, ALP activity and BGP content at 2.0 mmol/L added of P level were higher (*P* ≤ 0.024) than those at 0.0 mmol/L of added P level. The ERK1 silencing decreased (*P* < 0.05) the P retention rate of osteoblasts regardless of 0.0 or 2.0 mmol/L of added P level, but the reduced degree was greater at 2.0 mmol/L of added P level. As shown in Figures 5A–D, the ERK1 mRNA and protein expressions, as well as p-ERK1 protein expression were decreased (*P* ≤ 0.004) in ERK1-silenced cells. The P addition had no effect (*P* > 0.45) on the *ERK1* mRNA expression, but inhibited (*P* = 0.014) its protein expression, and upregulated (*P* = 0.015) the p-ERK1 protein expression. No interaction (*P* > 0.08) between cell type and added P level in ERK1 mRNA and protein expressions and p-ERK1 protein expression.

## Discussion

The hypothesis that either BMP2 or ERK1 silencing would affect P utilization in primary broiler osteoblasts has been supported by the results of the current study. In the present study, either BMP2 or ERK1 silencing significantly decreased the osteoblastic P retention rate, mineralization formation, ALP activity and BGP content, indicating that either BMP2 or ERK1 silencing suppressed P utilization, and thus either of them participated in regulating P utilization of primary broiler osteoblasts. The aforementioned new findings have never been reported before, and thus provided a new perspective and idea for developing feasible strategies for improving P utilization in the bone of broiler chickens.

The BMP and ERK1 signaling pathways play a crucial role in the maturation, differentiation and mineralization of osteoblasts (6–8, 17). The BMP family proteins, especially BMP2, are essential molecules for osteoblastic differentiation and key bone regulators (8). It was reported that the activation of BMP2 could further induce the phosphorylation of ERK1, to promote the osteogenic differentiation (1). Zhu et al. (10) reported that ERK1 inhibitor could suppress the ALP activity, bone mineralization and downregulate the expression of *BMP2* mRNA in mice bone mesenchymal stem cells. However, p-ERK1 was enhanced by neutralizing anti-BMP2/4 antibodies in hepatocytes, suggesting that BMP2 would exert its function via ERK1 pathway (18). In chickens, previous findings suggested that the BMP2 and ERK1 pathways would have a strong possibility to be involved in bone P utilization *in vitro* or *in vivo* (11, 12). As added P level increased, BMP2 mRNA expression increased, while ERK1 mRNA expression decreased in broiler tibia (12). Consistently, the *BMP2* mRNA expression increased linearly and quadratically, whereas the *ERK1* mRNA expression decreased linearly as added P level increased in primary osteoblasts cells (11). These results suggested that ERK1 expression might be inversely correlated with the BMP2 expression. In the present study, added P level had no effect on *BMP2* and *ERK1* mRNA expressions and BMP2 protein expressions, but inhibited ERK1 protein expression, and upregulated the p-ERK1 protein expression. Exact reasons and mechanisms for the above disparities remain unclear, and need to be further investigated in the future.

Osteoblasts play a major role in the bone formation and development. In broilers, bone P retention and bone mineralization-related parameters are sensitive indictors that reflect P utilization (12, 13, 15). The ALP and BGP have been demonstrated to participate in the process of hydroxyapatite formation and mineralization in osteoblasts (19). The ALP secreted by osteoblasts, is an enzyme hydrolyzing phosphate ester to provide inorganic P for hydroxyapatite formation (20, 21). Hence, the increased inorganic P resulting from the higher ALP activity implied that the bone tissue underwent more intense osteogenesis. The BGP is necessary for the osteogenic differentiation and is involved in bone density regulation to inhibit the development of osteoclasts. In addition, BGP regulates the hydroxyapatite crystals size and shape in bone growth (15). Our studies in broilers demonstrated that the ALP activities in serum and in the tibia, as well as BGP content in the tibia, decreased as added P level increased (15, 22, 23). Moreover, correlation analyses showed that the P utilization-related parameters were negatively associated with ALP activity and BGP content in broiler tibia (12, 15). In the present study, either BMP2 or ERK1 silencing significantly reduced the tibial osteoblastic ALP activity and BGP content. Added P level increased the tibial osteoblastic ALP activity and BGP content in the ERK1-silenced experiment, and the ALP activity was increased by P addition in the BMP2-silenced experiment. Therefore, the above results further suggested that both BMP2 and ERK1 would be involved in the regulation of the bone P utilization in broilers.

The BMP2 is known to induce the activation of ERK signaling molecules, thus to promote the osteoblastic differentiation and bone formation (10). In addition, P has been recognized as a particular signaling molecule that affects the expression of osteogenesis genes, including BMP2, ALP and BGP through the MAPK pathway (11, 12). However, no *in vitro* study has been reported on the effect of BMP2 or ERK1 silencing on P utilization of primary broiler osteoblasts. Our results indicated that BMP2 or ERK1 silencing suppressed P retention rate, mineralization formation, ALP activity and BGP content. The above findings will help us better understand the role of BMP2 or ERK1 in regulating P utilization of primary broiler osteoblasts and highlight their potential mechanisms.

## Conclusions

Either BMP2 or ERK1 silencing suppressed P utilization, and thus either of them was involved in regulating the P utilization in primary broiler osteoblasts.

## Data Availability Statement

The datasets presented in this study can be found in online repositories. The names of the repository/repositories and accession number(s) can be found in the article/supplementary material.

## Ethics Statement

The study protocol was approved by the Animal Care Committee of the Department of Animal Science and Technology of Yangzhou University, Yangzhou, China (permit number: SYXK (Su) 2021-0027) and conducted in accordance with the guidelines of the Animal Use Committee of the Chinese Ministry of Agriculture (Beijing, China).

## Author Contributions

YG and TL designed the experiments, analyzed the data, and wrote the original draft. YH, XC, BW, LZha, and LZhu performed the experiment. XL supervised and edited the thesis. All authors contributed to the article and approved the submitted version.

## Funding

This study was financed by the Key Program of the National Natural Science Foundation of China (Project no. 31630073; Yangzhou, P. R. China), the Natural Science Foundation of Jiangsu Province (Project no. BK20210809; Yangzhou, P. R. China), and Initiation Funds of Yangzhou University for Distinguished Scientists (Yangzhou, P. R. China).

## Conflict of Interest

The authors declare that the research was conducted in the absence of any commercial or financial relationships that could be construed as a potential conflict of interest.

## Publisher's Note

All claims expressed in this article are solely those of the authors and do not necessarily represent those of their affiliated organizations, or those of the publisher, the editors and the reviewers. Any product that may be evaluated in this article, or claim that may be made by its manufacturer, is not guaranteed or endorsed by the publisher.
